# Development and characterization of polymorphic microsatellite markers for *Periplaneta americana* (Blattodea: Blattidae)

**DOI:** 10.1093/jisesa/ieae096

**Published:** 2024-10-29

**Authors:** Li Lim, Abdul Hafiz Ab Majid

**Affiliations:** Household and Structural Urban Entomology Laboratory, Vector Control Research Unit, School of Biological Science, Universiti Sains Malaysia, Minden, Penang 11800, Malaysia; Household and Structural Urban Entomology Laboratory, Vector Control Research Unit, School of Biological Science, Universiti Sains Malaysia, Minden, Penang 11800, Malaysia

**Keywords:** microsatellite, American cockroach, marker, next-generation sequencing

## Abstract

The American cockroach, *Periplaneta americana* (Blattodea: Blattidae), is a prevalent urban pest with significant public health implications. This study aimed to develop and validate novel microsatellite markers to understand the genetic diversity and population genetic structure of *P. americana*. In this study, a total of 397,898 microsatellite markers were developed based on 24.6 million genomic DNA sequences. Twenty microsatellite markers were selected and amplified with varying numbers of alleles ranging from 0 to 35. Seven out of 20 markers were characterized for their polymorphism and amplification efficiency. The polymorphic information content (PIC) values of these markers were high (0.669 to 0.950) implying their effectiveness. These markers also revealed 7 to 35 alleles per locus across tested samples, highlighting their utility in assessing the extensive genetic variation within *Periplaneta americana* populations. These results provide insightful information that may be applied to the genetic analysis of the American cockroach population using the developed species-specific microsatellite marker.

## Introduction

The American cockroach, *Periplaneta americana* (Blattodea: Blattidae), is one of the most prevalent and persistent urban pests worldwide. Despite its common name, it is believed to have originated in Africa and has since spread globally, thriving particularly in warm, humid environments. This species is notable for its large size, rapid movement, and adaptability to various habitats, including human dwellings, which make it a significant vector for allergens and pathogens (Chen et al. 2015, Li et al. 2018).

Effective management and control of *P. americana* populations are crucial for public health and hygiene. However, understanding the genetic structure and population dynamics of this species is essential for developing targeted and sustainable pest control strategies. Molecular markers, such as microsatellites, have proven to be invaluable tools in population genetics studies due to their high polymorphism, codominant inheritance, and reproducibility (Bruford and Wayne 1993).

Microsatellite markers or simple sequence repeats, consist of short, tandemly repeated DNA sequences found throughout the genome (Bhargava and Fuentes 2010). These markers are highly variable among individuals, making them ideal for assessing genetic diversity, population structure, gene flow, and relatedness (Bruford and Wayne 1993, Buschiazzo and Gemmell 2006). Despite their utility, developing and characterizing microsatellite markers for *P. americana* have been limited.

This study reported the development and validation of novel microsatellite markers for *P. americana*. By employing next-generation sequencing technologies, a robust set of polymorphic microsatellite loci was identified. These markers will provide a valuable resource for future genetic studies and contribute to a deeper understanding of the population genetics and evolutionary biology of this pervasive pest.

## Materials and Methods

### Sample Collection and DNA Extraction

Cockroaches were collected from sewers at 4 locations (abbreviated as A, B, C, and D; Table 1) within the Hostel Bakti at Universiti Sains Malaysia on May 24 to 25, 2023.

**Table 1.** Location and number of samples (*Periplaneta americana*) collected

Location	Assigned code	Latitude	Longitude	Number of samples
Hostel Bakti Manhole 1	A	5°21ʹ26.64″N	100°17ʹ59.64″E	35
Hostel Bakti Manhole 2	B	5°21ʹ24.48″N	100°17ʹ58.56″E	40
Hostel Bakti Manhole 3	C	5°21ʹ23.04″N	100°17ʹ59.28″E	17
Hostel Bakti Manhole 4	D	5°21ʹ36.72″N	100°18ʹ11.16″E	11

One sample was randomly selected, and its DNA was extracted from the leg using the HiYield Plus Genomic DNA Mini Kit (Blood/Tissue/Cultured Cells; Real Biotech Corp., Taipei, Taiwan), following the manufacturer’s protocol. A total of 100 μL of DNA was collected and validated using a NanoDrop 2000c (Thermo Fisher Scientific, MA). Illumina DNA paired-end libraries were constructed according to the standard protocol provided by Illumina (San Diego, CA, USA), with short-insert sizes (300 bp). Sequencing was then performed on the MiSeq platform (Illumina). Additionally, a total of 40 samples (10 from each location) were selected for DNA extraction to test the designed markers.

### Microsatellite Marker Design

Microsatellite markers were identified from the draft genome sequencing dataset of *P. americana* by screening with OSTRFPD v0.01 (Mathema et al. 2019), using a minimum of 8 repeats for motifs ranging from 2 to 6 nucleotides. Primer pairs flanking the isolated microsatellites were designed using Primer3 (Untergasser et al. 2012) with default settings. The size range for the primers was set to 17 to 26 bp, with a melting temperature of 58 to 63 °C and a GC content of 20% to 80%. Other criteria included an amplicon length of 100 to 300 bp and the absence of secondary structures.

### Microsatellite Marker Validation

Twenty microsatellite markers (Table 2) were selected from the designed markers (Supplementary Table S1) for the polymorphism analysis based on the length (number of repeats) and type of repeat motif. The microsatellite markers were used to amplify 40 individual *P. americana* genomes. The PCR was performed using a Thermal Cycler (TaKaRa, Japan). The PCR mixture contained 12.5 μL of Master mix consisting of green buffer (NX, Nuclix Biosolution, Malaysia), 0.5 μL of 5 mM of each primer, 5 mL DNA template, and sterile cold distilled water made up to 25 μL. PCR cycles consisted of 40 cycles of denaturing at 94 °C for 30 s, annealing at varied temperatures (shown in Table 2) for 30 s, extension at 75 °C for 45 s, and a final extension at 75 °C for 5 min.

**Table 2.** Information about 20 microsatellites develop from *Periplaneta americana*

Locus	Repeat motif	Primer sequences	Tm (°C)^a^	N_A_^b^
PADi1	(AC)_53_	F: ATGTGGGAAGGACCTGCACT R: TTTCTACGAAACTCGAGGGC	56	20
PATri1	(ATG)_50_	F: CATGTGGGAAATAAATACCTGAAA R: TGCTTTGTTCGGAAGTGTTG	50	5
PATri2	(AAC)39	F: TCCTCCGTGTTCATGTTGAC R: TGGAAACACGTACTGAGGTGA	56	1
PATri3	(ACT)_39_	F: TTTGAACTGGAATGAACGGC R: TCCATTGAAGCCAGCCATA	54	9
PATri4	(AAT)_28_	F: AAGGAACCTTGGCGAGTTCT R: TCTTTACGAACCGCCTCAAT	55	1
PATetra1	(AGAT)_37_	F: AGACCGTAACAAGCCACCAG R: GGTGACAGAGGGCACTCCTA	59	3
PATetra2	(ACCT)_37_	F: CGTCCAAATGTATAAATCGTCG R: GCAAGAATTAGCACAATCGGT	51	3
PATetra3	(ACAG)_37_	F: AAGAGCACGTTCTACGTTTATGA R: TGTGACTGAGGGAACCATGA	54	20
PATetra4	(ATCC)_37_	F: TCTCAGTTTCTGTAAGCCGGA R: ATCCTCATCCTCACGACACA	56	2
PATetra5	(CAAG)_37_	F: CTTACGGCGTTAACTCTGCC R: GCACTGTTTGCTCGTGATACTT	56	3
PATetra6	(AACC)_37_	F: AGGGCCGATAAGCTGATACA R: ACACACGATCTCGCACAAAG	56	35
PATetra7	(ACAT)_35_	F: AAGGGCACAAATACCCACAA R: TTTAAATCACTCGCCCACTTT	52	0
PATetra8	(ACTC)_27_	F: TGTACTTGAGTACGTCCTCTTTCAA R: GAAGATTTCATTAAGAAAGCGAACA	51	2
PAPenta1	(ATCCG)_30_	F: TGGTTGTGAAACTTGGACTCTC R: TCACTACATTCGTTTCATGGTTG	53	3
PAPenta2	(ACGAT)_30_	F: AACGTCTGATTCACATTGGG R: AGGATACGATATCTTAGGCCATTT	53	1
PAPenta3	(ACCAG)_30_	F: CACAAAGGAGACGCATATAGGTA R: CAGGACGATTTAGGAGTAAAGGG	54	13
PAPenta4	(ATCGG)_30_	F: AGCCTAAAGCTAAGGACGCC R: TCAGGCTACAAATCCAAGGG	56	2
PAPenta5	(CAGAG)_29_	F: CAAATATGTGGCTTCCGGTC R: GTTTCTCATTACAACGCTCTGC	55	1
PAPenta6	(AACCT)_29_	F: GCTTTCATTTGAAATCTTCCCA R: AAGCTTGGTTTCAGACTTCCC	51	1
PAHexa1	(ACTGCT)24	F: TACTGTTCTGAAGCGGCCTT R: CACTTGGTATCAATCAACCAACA	53	7

^a^Tm (°C)—annealing temperature.

^b^N_A—_number of alleles observed.

Fragment analysis was carried out using the Fragment Analyzer Automated CE System (Agilent Technologies, CA). The number of alleles was scored with the Prosize 3.0 software package (Agilent Technologies, CA). Out of the 20 microsatellite markers, 7 were selected for further validation based on the type of motif and amplification effectiveness. The selected microsatellite markers are dinucleotide motif, PADi1; trinucleotide motif, PATri3; tetranucleotide motif, PATetra3 and PATetra6; pentanucleotide motif, PAPenta3 and PAPenta4; and hexanucleotide motif, PAHexa1. Mononucleotide microsatellite markers were excluded from primer selection due to the high error rate during amplification (Shinde 2003, Baptiste et al. 2015).

Micro-Checker v2.2.0.3 (Van Oosterhout et al. 2004) was used to analyze the fragment analysis results, specifically examining the observed and expected null allele frequencies to detect errors such as amplification failure, stuttering, or large allele dropout during PCR. Allele frequency analysis, including observed and expected heterozygosity, the number of alleles, and the polymorphic information content (PIC), was conducted using Cervus v3.0.7 (Kalinowski et al. 2007). Hardy–Weinberg equilibrium (HWE) was evaluated in GENEPOP (Rousset 2008) while F_ST_ value was (Weir and Cockerham 1984) computed using GenAiEX, version 6.5 (Peakall and Smouse 2006).

## Result and Discussion

Microsatellites were screened from 24.6 million raw sequence reads. The identified microsatellites were distributed as follows: 53.09% mononucleotide, 4.84% dinucleotide, 24.39% trinucleotide, 13.48% tetranucleotide, 4.03% pentanucleotide, and 0.16% hexanucleotide repeats. From these, 397,898 primer pairs flanking the screened microsatellites were designed, as detailed in Supplementary Table S1.

From the myriad number of designed primers, 20 primers were selected, with most of them successfully genotyping 40 individual *P. americana* specimens from 4 populations with various allele counts (ranging from 0 to 35 alleles), as shown in Table 2. Then, 7 markers that showed higher numbers of alleles were selected for further validation. These markers demonstrated high genetic diversity, producing 7 to 35 alleles per locus among samples from 4 locations (Table 3). High genetic diversity suggests that many different alleles exist within the *P. americana* populations, indicating a diverse genetic makeup. Fragment analysis also revealed no scoring errors for these markers due to stuttering peaks or large allele dropouts.

**Table 3.** Characteristics of 7 microsatellite DNA loci developed for *Periplaneta americana* and screened for a total of 40 specimens collected from 4 locations of sewer system in USM: results from software Micro-checker (stuttering, allele dropout, and present of null alleles), software Cervus (number of alleles observed [N_A_], average expected [H_E_] and observed [H_O_] heterozygosities, and PIC value), and GenAiEX (F_ST_)

Locus	Micro-checker			Cervus				F_ST_ (*P*-value)
	Stuttering	Allele dropout	Present of null alleles	N_A_	H_E_	H_O_	PIC	
PADi1	No	No	Yes	20	0.922	0.225	0.904	0.117 (0.001)
PATri3	No	No	Yes	9	0.803	0.000	0.765	
PATetra3	No	No	Yes	20	0.936	0.525	0.920	
PATetra6	No	No	Yes	35	0.964	0.325	0.950	
PAPenta3	No	No	Yes	13	0.906	0.050	0.886	
PAPenta4	No	No	Yes	9	0.838	0.000	0.803	
PAHexa1	No	No	No	7	0.709	0.125	0.669	

The PIC values ranged from 0.669 to 0.950, indicating that the microsatellite markers possess the desired polymorphism characteristics (Botstein et al. 1980). These high PIC values further confirm the utility of the markers in differentiating between individuals and assessing population structure.

The observed heterozygous (H_o_) frequencies differed from those expected, indicating deviations of HWE in all tested populations (Table 3). HWE analysis (Supplementary Table S1) supported the previous indication and revealed significant deviations across populations in all tested loci. For example, in population C, the Fis estimates for loci “PADi1” and “PATetra3” are 0.8750 and −0.1921, respectively. The positive Fis value (0.8750) at locus “PADi1” indicates an excess of homozygotes, which could be attributed to several factors, such as inbreeding, where individuals within this population might be mating with relatives, leading to higher homozygosity at this locus (Waller and Keller 2020). Additionally, population subdivision could result in limited gene flow within subpopulations or microhabitats, causing an excess of homozygotes (Whitlock 2002, Garnier-Géré and Chikhi 2013). Positive selection for homozygous genotypes at “PADi1” might also be occurring, possibly due to a local environmental factor that provides a selective advantage to those genotypes.

Conversely, the negative Fis value of −0.1921 at locus “PATetra3” indicates a deficit of homozygotes, which might be due to a heterozygote advantage, where heterozygous individuals have a selective advantage, increasing the frequency of heterozygotes. An increased proportion of heterozygotes could also be due to assortative mating, where individuals preferentially mate with genetically dissimilar partners (Hedrick 2016). Furthermore, recent gene flow from different populations with differing allele frequencies could increase heterozygosity at this locus, particularly if the incoming population has a high frequency of different alleles (Selander and Kaufman 1973).

F_ST_ values can range from 0 to 1, with high F_ST_ implying considerable differentiation among populations. The observed F_ST_ value of 0.117 is close to 0, indicating low differentiation among the populations studied. This means that despite the high genetic diversity within each population (as indicated by the microsatellite markers), there is relatively little genetic differentiation between populations. This low F_ST_ value suggests that there is substantial gene flow among populations, which contributes to maintaining genetic similarity. The populations are interbreeding and exchanging genetic material, which may enhance their ability to cope with environmental changes and pressures, particularly in urban settings where they are commonly found (Morjan and Rieseberg 2004).

## Supplementary Material

ieae096_suppl_Supplementary_Tables_S1
